# Genomic structure of stx2i genes and prophages determined in Shiga toxin-producing Escherichia coli isolated from food, animal and human samples in Germany

**DOI:** 10.1099/mgen.0.001841

**Published:** 2026-09-30

**Authors:** Maria Borowiak, Carlus Deneke, Christina Lang, Andre Göhler, Angelika Fruth, Elisabeth Schuh, Michaela Projahn

**Affiliations:** 1 Department of Biological Safety, German Federal Institute for Risk Assessment, National Study Centre for Sequencing in Risk Assessment, Diedersdorfer Weg 1, 12277 Berlin, Germany; 2 Robert Koch Institute, Division of Enteropathogenic Bacteria and Legionella, National Reference Centre for Salmonella and other Bacterial Enterics, Burgstraße 37, 38855 Wernigerode, Germany; 3 Department of Biological Safety, German Federal Institute for Risk Assessment, National Reference Laboratory for Escherichia coli including VTEC, Diedersdorfer Weg 1, 12277 Berlin, Germany

**Keywords:** phage, Shiga toxin, Shiga toxin-producing *Escherichia coli* (STEC), *stx2i*

## Abstract

Shiga toxin-producing *Escherichia coli* (STEC) are important foodborne pathogens and a threat to human health. Their main virulence factor, the Shiga toxin (Stx), is encoded in two subunit genes (*stxA* and *stxB*) and occurs in various subtypes. Here, we report the occurrence of strains harbouring the new subtype *stx2i*. Isolates from food (*n*=8), animal (*n*=3) and human (*n*=2) samples were subjected to whole-genome sequencing for detailed molecular analyses. All strains harboured an identical *stx2i* variant independent of the genoserotype and differed from the first detected variant in 2016. In five of the strains, inactive *stx* operons were determined due to a transposon insertion in the *stxA2i* subunit gene. *Stx* phages and insertion sites were comparable between the strains of the same genoserotype. As the *stx2i* variant was also identified in a human clinical sample, we developed a specific PCR, as there are possible cross-reactions with other typing methods currently used in routine diagnostics. In total, our data show that STEC strains of this specific *stx2i* variant occur in various matrices of food, animals and humans and that there might be an underdetected number of strains due to insufficient typing methods.

**Impact Statement**

In our study, we identified various Shiga toxin-producing *Escherichia coli* (STEC) strains harbouring the Shiga toxin subtype Stx2i, which was assigned in 2016 to a strain isolated in 2005. Until then, only one additional report on the occurrence of STEC harbouring *stx2i* genes was available. Here, we found that *stx2i* is more widespread than previously thought, and it was found in various matrices, including human samples. Furthermore, we identified genetic variations in the *stx2i* operon due to transposon insertions. Based on our findings, we hypothesized that *stx2i* strains are underdiagnosed due to the lack of a specific PCR method. We therefore developed a PCR which detects this subtype and has no cross-reactivity to known *stx* subtypes.

## Data Summary

Public data on *stx2i* genes were available from the National Center for Biotechnology Information under GenBank number FN252457.1 (CB10366 [1]) and BioSample SAMEA4964526 (STEC from oyster 663_811_23 [2]), respectively. Sequencing data generated during this study were deposited under NCBI BioProject Accession PRJNA1309617.

The authors confirm that all supporting data, code and protocols have been provided within the article or through supplementary data files.

## Introduction

Shiga toxin-producing *Escherichia coli* (STEC) are enteric pathogens that are a risk to human health as they can cause diarrhoea and the haemolytic uraemic syndrome [3]. They are naturally occurring in the gastrointestinal tracts of cattle and small and wild ruminants [4] but can also lead to oedema disease in pigs [5]. Human infections are mainly caused by the consumption of contaminated foodstuff like beef and dairy products [6].

The most important virulence factor of STEC is the Shiga toxin, which belongs to the AB_5_ toxin class [7]. There are two main types of Shiga toxins, Stx1 and Stx2, which have an approximately aa identity of 50–60% and differ in their pathogenic effects in humans [8–10]. Currently, there are three known subtypes for Stx1 toxins (a, c and d) and 15 subtypes for Stx2 toxins (a–o) in *E. coli* [10–13]. The most frequent Stx subtype involved in porcine oedema disease is the Stx2e subtype [14]. Shiga toxins are encoded in an *stx* operon consisting of the genes for the two subunits, A and B. These operons are located in the bacterial chromosomes within prophages or lysogenic phages, mostly having a lambdoid genome structure [15]. Lysogenic phages can enter the lytic cycle due to environmental stress or other exogenous factors. With that, they can spread the *stx* genes via horizontal gene transfer across other *E. coli* strains [16].

STEC harbouring the *stx2i* subtype was first isolated from a shrimp in 2005 [1]. Since then, this subtype has been detected once in STEC from an oyster [2] but has not yet been isolated from human clinical specimens. In our study, we analysed STEC strains from our collections in Germany (food, animal and human isolates) on the occurrence of the *stx2i* subtype and provide insights into the genomic structure and genetic rearrangements in *stx2i* genes and prophages from 12 *stx2i*-positive STEC strains. We also developed an *stx2i*-specific PCR for accurate detection of this subtype within diagnostic workflows, as some current subtyping PCRs show cross-reactions to the new variants due to sequence identities in the primer binding sites.

## Methods

### STEC strains

The study comprises 13 strains from food (*n*=8), animal (*n*=3) and human (*n*=2) samples (Table 1). Strains from food and animal samples were isolated by federal state laboratories as part of the German food control and the zoonosis monitoring programme and sent to the National Reference Laboratory for *Escherichia coli* including VTEC (NRL-*E. coli*) for confirmation and further analyses. Strains BfR-EC-18545 and BfR-EC-18937 were isolated from minced meat from beef and pork, whereas strains BfR-EC-18848, BfR-EC-18946, BfR-EC-19518, BfR-EC-19994 and BfR-EC-20407 were detected in frozen meat of lamb or sheep, respectively. Strains BfR-EC-19583, BfR-EC-19621 and BfR-EC-19736 were isolated from swine faeces samples taken at the slaughterhouse level. Strain BfR-EC-10366 (CB10366) with an already published *stx2i* gene (GenBank: FN252457 [1]) was isolated from a shrimp sample. Strains 24-06088 and 25-01906 were isolated at the National Reference Centre for *Salmonella* and other Bacterial Enterics from human clinical samples (Table 1). Strain BfR-EC-10366 (CB10366) with an already published *stx2i* gene was whole-genome sequenced during this study. Genomic data for the additional strain 663_811_23 (STEC from oyster) were downloaded from NCBI for genomic comparisons as a reference. Shiga toxin production for strains from food samples and animal faeces was tested using the Ridascreen^®^ Verotoxin ELISA (R-Biopharm^®^ AG, Darmstadt, Germany) according to the manufacturer’s instructions.

**Table 1.** Overview of STEC strains used in the study

Strain	Year of isolation	Isolation source	Genoserotype	Warwick MLST	Additional virulence factors	NCBI BioSample (reference)
BfR-EC-10366 (CB10366)	2005	Shrimp	O9:H4	ST10830	*sta1*, *stb*	SAMN50749559 [1]
663_811_23	2016	Oyster (Norway)	O9:H10	ST88		SAMEA4964526 [2]
BfR-EC-18848	2020	Frozen meat from leg of lamb/sheep (food chain)	Onovel8:H21	ST155		SAMN50749561#
BfR-EC-18946	2021	Frozen meat from leg of lamb/sheep (food import)	O30:H25	ST8660		SAMN50749563#
BfR-EC-19518	2022	Frozen meat from leg of lamb/sheep (food chain)	O100:H30	ST993		SAMN50749564#
BfR-EC-19994	2023	Frozen meat from leg of lamb/sheep (food chain)	O100:H30	ST993		SAMN50749568#
BfR-EC-20407	2024	Mixed minced meat from beef and lamb/sheep (food chain)	O100:H30	ST993		SAMN50749569#
24-06088	2024	Human	O9:H30	ST1294		SAMN57320383#
25-01906	2025	Human	Onovel8:H21	ST155		SAMN57320384#
BfR-EC-18545*	2020	Mixed minced meat from beef and pork (food chain)	O8:H9	ST23	*stx2e*	SAMN50749560#
BfR-EC-18937*	2021	Mixed minced meat from beef and pork (food chain)	O8:H9	ST23	*stx2e*	SAMN50749562#
BfR-EC-19583*	2023	Faeces, swine (slaughter house)	O8:H9	Unknown (new icd)		SAMN50749565#
BfR-EC-19621*	2023	Faeces, swine (slaughter house)	O8:H9	ST23	*2x stx2e*	SAMN50749566#
BfR-EC-19736*	2023	Faeces, swine (slaughter house)	O8:H9	ST23		SAMN50749567#

* Strains with insertions in the *stx* operon.

#T﻿his study.

### DNA isolation and whole genomic sequencing

Whole genomic DNA was isolated using the PureLink^®^ Genomic DNA Mini Kit (Invitrogen, USA) according to the manufacturer’s instructions or by glass bead extraction [17]. Short-read sequencing libraries were prepared with the Illumina DNA Prep (M) Tagmentation Kit (Illumina, San Diego, USA) or the Nextera XT DNA Library Preparation Kit (Illumina, San Diego, CA, USA), and paired-end sequencing was performed on an Illumina MiSeq benchtop sequencer [MiSeq Reagent Kit v3 (600 cycles)], NextSeq500 [NextSeq 500/550 Mid Output Kit v2.5 (300 cycles)] or NextSeq2000 (300 cycles). For nine selected strains, DNA libraries for Oxford Nanopore were prepared using Rapid barcoding kits (SQK-RBK004, SQK-RBK110-96 or SQK-RBK114-96). Sequencing was performed using R9.4.1 or R10.4.1 flow cells on MinION Mk1B or Mk1C instruments. For more details, see Table S1 (available in the online Supplementary Material). DNA isolation for PCR testing was done from pure cultures using the boiling method with an incubation of 15 min at 95 °C.

### Bioinformatic analyses

Raw Illumina reads were trimmed and *de novo* assembled using the AQUAMIS pipeline [18]. fastq files of sample 663_811_23 [2] were downloaded from the NCBI database (BioSample: SAMEA4964526), and the AQUAMIS pipeline was applied for *de novo* assembly of the genome. The genomes of nine strains from the NRL-*E. coli* collection were assembled using a hybrid approach, combining Illumina and Oxford Nanopore sequence data with the Microbial Long Read Assembly pipeline (MiLongA, https://gitlab.com/bfr_bioinformatics/milonga), which utilizes the flye assembler [19] with subsequent pilon polishing [20] or the unicycler assembler [21, 22]. For more details, see Table S1. Genome completeness was assessed using CheckM [23]. Strain characterization was done using BakCharak (https://gitlab.com/bfr_bioinformatics/bakcharak, v 3.0.3 [24]) which implements determination of the genoserotype based on the CGE SeroTypeFinder 2.0 [25] and EcOH [26], as well as the MLST typing tool mlst (https://github.com/tseemann/mlst). Sequence annotation was done using bakta v1.9.3 [27]. For prediction of *stx* phage regions, the PHASTER web tool (https://phaster.ca/ [28]) was used. Multiple alignments and sequence comparisons of *stx* genes and STEC phages were done with Geneious Prime^®^ (version 2020.2.2., Biomatters Ltd.) and Easyfig version 2.2.5 [29]. Additional virulence factors were determined using BioNumerics (Version 7.6.3, AppliedMath).

### *Stx2i*-specific PCR

PCR primer *stx2i*-F1 (5′ GGCTGGATTTGTTAATACGACAACA 3′) and *stx2i*-R5 (5′ TGACTGAGACTTTCTGTTCAGAAAG 3′) were designed with the OligoPerfect Primer Designer tool (https://www.thermofisher.com/de/de/home/life-science/oligonucleotides-primers-probes-genes/custom-dna-oligos/oligo-design-tools.html) and Geneious Prime^®^ (version 2020.2.2., Biomatters Ltd.) using the *stx2i* reference sequence (FN252457) and a set of reference sequences comprising all known *stx2* subtypes [10, 11, 30–32]. The total PCR reaction mixture of 25 µl contained 5 µl of MyTaq Red Mastermix (Bioline, USA), 1 µl of each primer (10 µM), 0.15 µl of Taq Polymerase, 1 µl of the sample DNA and 16.85 µl of PCR water. PCR running conditions were as follows: 94 °C for 2 min initial denaturation, 30 cycles of 94 °C for 1 min, 64 °C for 30 s, 72 °C for 1 min and a final elongation step of 72 °C for 5 min. For specificity testing, PCR was performed against known *stx2* types using boiled DNA extracts from strains from routine diagnostics [33] and *E. coli* strains transformed with plasmids encoding toxoid sequences (enzymatically inactive A subunit) [34].

## Results and discussion

### Characterization of STEC strains

The 13 strains from food, animal and human samples were investigated concerning genoserotype and MLST type, as well as their respective *stx* genes using whole genomic sequences, and compared to already published data. All food and animal strains were subjected to Illumina and ONT sequencing to receive high-quality hybrid genomes. Strains BfR-EC-19994 and BfR-EC-20407 were only subjected to Illumina sequencing, as they showed the same basic characteristics like serovar, MLST and *stx* gene type as BfR-EC-19518. In total, the 13 investigated strains belong to 6 different genoserotypes and 6 MLST sequence types (Table 1). Strain BfR-EC-19518 belongs to a yet-unknown MLST ST. All strains are non-O157 strains; however, several studies showed that non-O157 strains are of importance because of their high prevalence in foods and that they can also cause severe infections in humans [35–40]. Eight strains from food and human samples, including BfR-EC-10366 (CB10366), carried complete *stx2i* genes, whereas in five strains, an insertion in the *stx2i* operon was detected (see 7.1). Those strains were isolated from food and animal faeces. Until now, only two STEC strains harbouring the *stx2i* gene variant were published. Strain BfR-EC-10366 (CB10366) was isolated from a shrimp in 2005 [1], and strain 663_811_23 was isolated from an oyster in 2016 [2]. Our strains were mainly isolated from meat from lamb/sheep, minced meat from pork or pig faeces which differs from the isolation source of the previously isolated strains. The detection of further STEC with the *stx2i* subtype in other animals and foods suggests that this gene variant is more widely distributed than previously thought. A possible explanation might be the lack of a specific detection method or the misidentification of this variant. Also, to the best of our knowledge, we report here the first *stx2i* strains isolated from human clinical samples, indicating an importance for human health, especially as one of the human strains (25-01906) showed the same genoserotype and MLST type as a food strain (BfR-EC-18848). In addition to the *stx2i* gene, the *stx2e* gene was also identified in strains BfR-EC-18545, BfR-EC-18937 and BfR-EC-19621, which is commonly found in STEC isolates from pigs and pork [14]. Strain BfR-EC-10366 was additionally positive for *sta1* and *stb* coding for heat-stable toxins; however, those virulence genes were not identified in the newly detected strains. Moreover, none of the strains carried the virulence gene *eae*, yet the human strains caused distinct clinical symptoms. The STEC strains were also tested concerning Shiga toxin production. All but BfR-EC-19583 and BfR-EC-19736 showed positive results in the Ridascreen ELISA, which is most likely due to the insertion within the *stx2i* operon. Positive results in the ELISA were detected for strains BfR-EC-18545, BfR-EC-18937 and BfR-EC-19621, which also show the insertion in the *stx2i* genes but harbour the additional *stx2e* gene.

Taken together, our strains can be divided into two groups: first, eight strains with complete *stx2i* genes isolated from meat of lamb/sheep, shrimp and human samples of genoserotypes O100:H30, O30:H25, O9:H4, O9:H30 and Onovel8:H21; second, five strains of genoserotype O8:H9 harbouring insertions in the *stx2i* operon which were isolated from pig faeces or mixed minced meat with pork.

### Comparison of *stx2i* gene variants

For more detailed analyses, the sequences of *stx2i* genes from the seven strains BfR-EC-18848, BfR-EC-18946, BfR-EC-19518, BfR-EC-19994, BfR-EC-20407, 24-06088 and 25-01906 were extracted from Illumina or flye/unicycler assemblies (if available) and compared to the reference sequence of BfR-EC-10366 (GenBank accession FN252457.1), as well as the already published *stx2i* sequence of the oyster strain 663_811_23 [2] using Geneious Prime^®^ (Fig. 1). The multiple alignment showed 100% identity of *stx2i* variants of our strains to strain 663_811_23. Comparison to the reference strain BfR-EC-10366 revealed a nucleotide identity of the *stx2i* operon of 99.59% with 5 mutations in the first 27 nucleotides of the *stxA2* subunit. Three out of the five mutations lead to aa changes in the protein sequence (Fig. S2). Furthermore, there are differences in the tRNA gene sequences downstream of the *stx2i* genes. BfR-EC-10366 has a complete sequence structure of tRNA-Ile-tRNA-Arg-tRNA-Arg, whereas all other strains show genetic variations with only two annotated tRNA genes, tRNA-pseudo-tRNA-Arg. The sequence comparison of the *stx2i* gene variants shows that the 663_811_23 variant is more common than the variant of strain BfR-EC-10366, which was identified first. Furthermore, the strains BfR-EC-18848, BfR-EC-18946, BfR-EC-19518, BfR-EC-19994, BfR-EC-20407, 24-06088 and 25-01906 belong to five different genoserotypes. This suggests that the *stx2i* prophage, upon induction/reactivation, can spread via horizontal gene transfer (transduction).

**Fig. 1.**
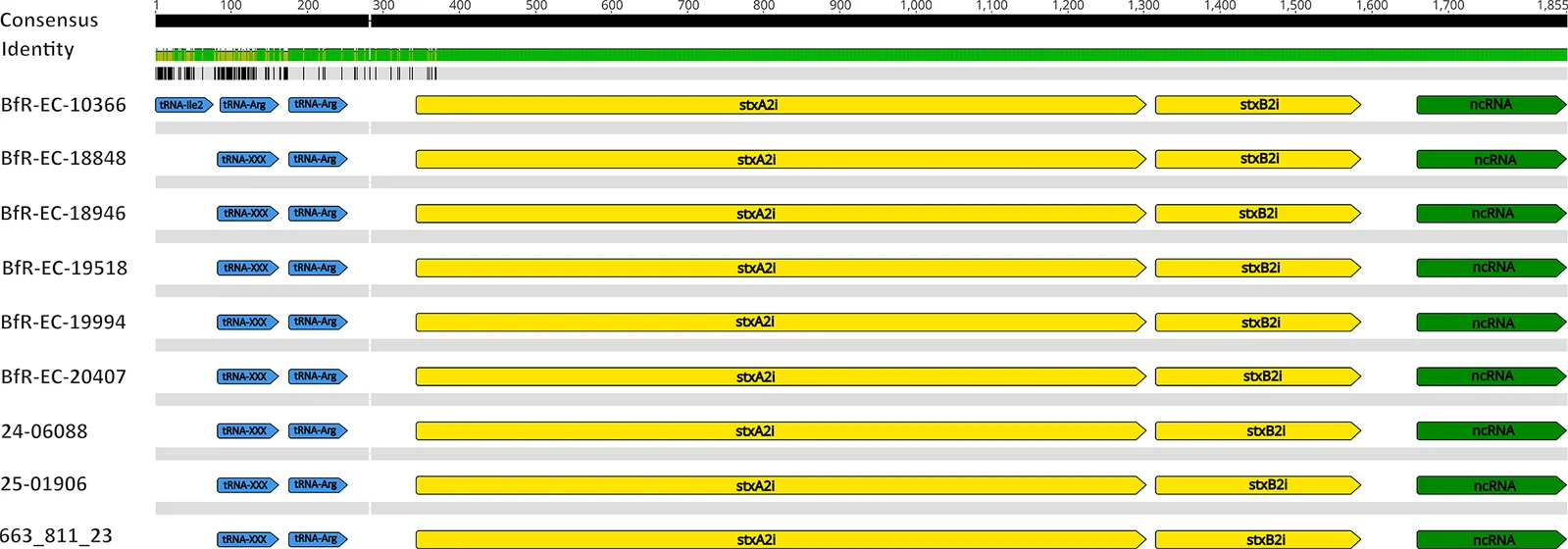
Sequence alignment of the *stx2i* genes showing consensus and identity blocks, as well as differences in nucleotides between the sequences of the six strains BfR-EC-18848, BfR-EC-18946, BfR-EC-19518, BfR-EC-19994, BfR-EC-20407, 24-06088 and 25-01906, as well as the two references. Yellow: *stx* gene subunits A and B; blue: tRNAs; tRNA-XXX: aa of a tRNA is unknown.

Strains BfR-EC-18545, BfR-EC-18937, BfR-EC-19583, BfR-EC-19621 and BfR-EC-19736 have a non-functional *stx2i* operon due to a transposase integration in the *stxA2* subunit (Fig. 2a). The *stx2i* gene sequence is identical to those strains harbouring the complete *stx2i* gene 663_811_23 variant mentioned above (Fig. 1). Such transposase insertions have been previously detected also in other *stx2* variants [41–44]. In the case of the *stx2i* strains, the insertions were found in the gene coding for the A subunit instead of the B subunit gene (Fig. 2a). To further investigate this, we compared the IS2 transposase insertion of a previously published strain BfR-EC-18960 [41] with those detected in the *stx2i* strains. It was found that the detected IS2 transposons are 99.7% identical according to BLASTn and also show nearly identical IRL and IRR sites (Fig. 2b). This shows that IS2 transposases are integrating into different *stx2* subtype genes and most likely contribute to rearrangements in STEC genomes and might facilitate the evolvement of new *stx2* subtypes. Strains BfR-EC-19583 and BfR-EC-19736 were proven to be negative in the Shiga toxin ELISA, showing the inactivation of the Stx2i protein due to the transposase insertion.

**Fig. 2.**
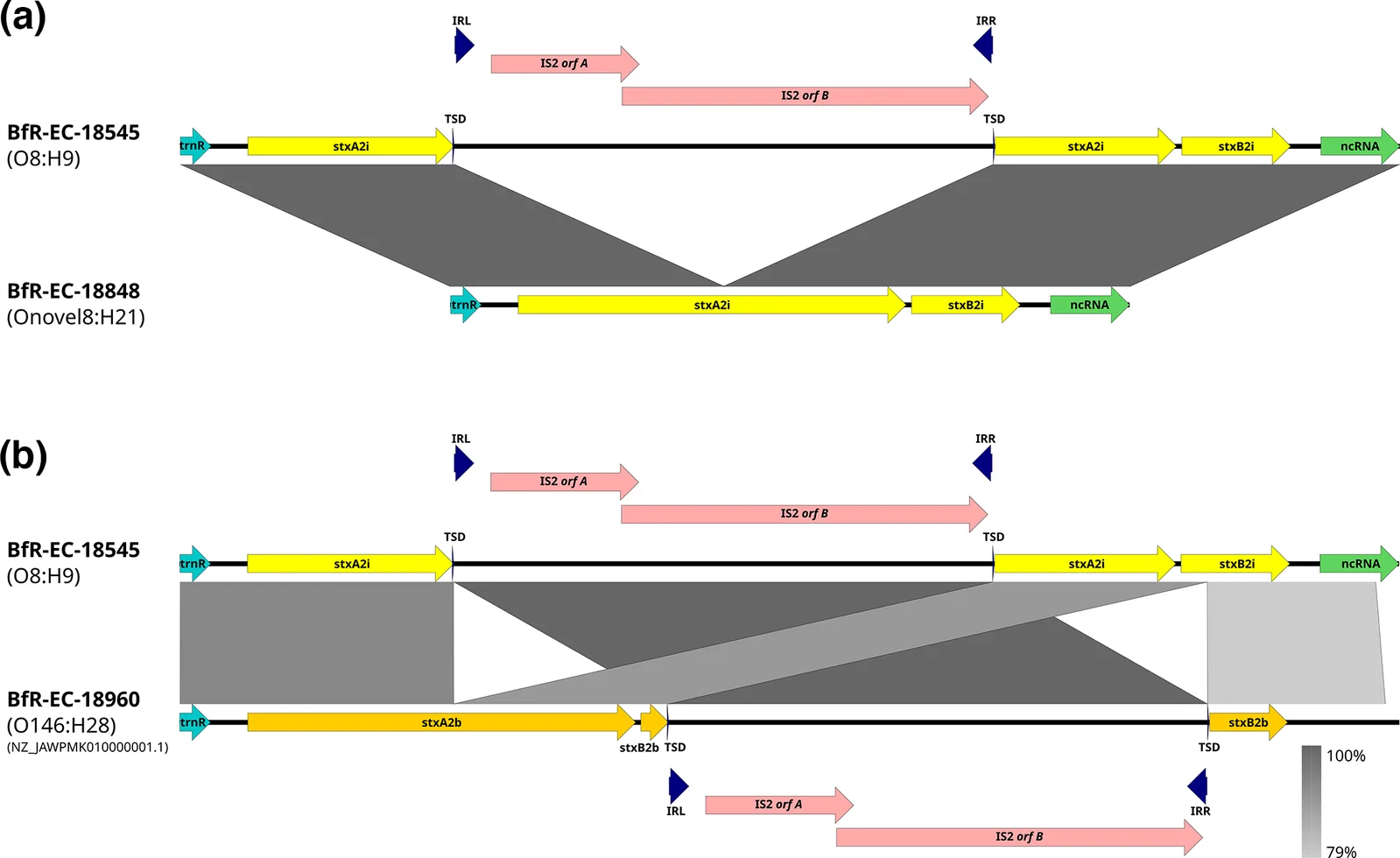
(a) Comparison of the *stx2i* region between BfR-EC-18848 (containing a complete *stx2i* gene region) and BfR-EC-18545 (*stxA2i* subunit disrupted by the IS2 transposon integration). (b) Comparison between BfR-EC-18545 (*stxA2i* subunit disrupted by the IS2 transposon integration) and BfR-EC-18960 (*stxB2b* subunit disrupted by IS2 transposon integration [41]). Genomic regions were visualized in Easyfig 2.2.5 and finalized in Inkscape version 1.3. Yellow: *stx* gene subunits A and B; blue: tRNAs; green: ncRNA; red: IS elements; greyscale: sequence identity; IRL/IRR=inverted repeats.

### Analyses of *stx2i* phages and integration sites

In the following, the nine strains that were subjected to long-read sequencing and for which closed chromosome sequences are available were examined in detail for the *stx*-phage integration sites. For visualization, the starting point of all genomes was set to the *dnaA* gene. *Stx*-phage regions were predicted using PHASTER. A total of three different *stx2i* integration sites were observed (Fig. 3). BfR-EC-19621, BfR-EC-18545 and BfR-EC-18937 showed the integration of one or two additional *stx2e* phages.

**Fig. 3.**
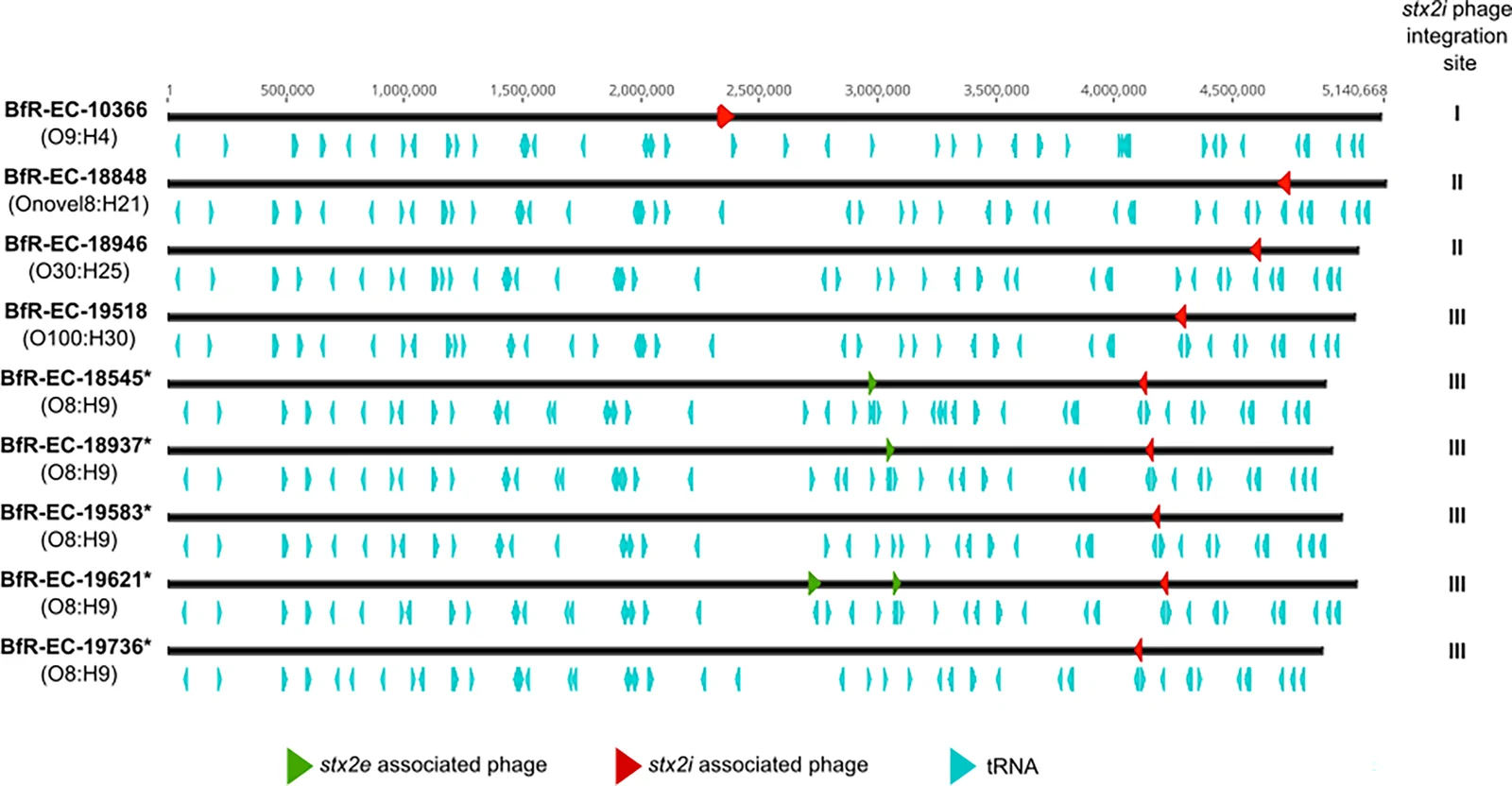
*stx2i* and *stx2e* gene and phage locations in the nine selected strains investigated using long-read sequencing. Complete and circularized chromosomes were visualized in Geneious Prime, and the figure was finalized in Inkscape version 1.3. tRNA arrays were visualized for better orientation in the genome. Strains with a transposon-interrupted *stxA2i* subunit are marked with an asterisk (*).

To investigate the *stx2i* phages in more detail, the respective predicted phage regions were compared using Easyfig (Fig. 4). Since all five strains carrying *stxA2i* with IS2 transposon integration had highly similar phage regions, only strain BfR-EC-18545 was selected as a representative strain for this group. The results for strain BfR-EC-10366 (*stx2i* phage integration site I) show that the predicted phage region is highly interspersed with transposons that are integrated into each other. A total of 11 complete and 3 incomplete IS-element or transposon insertions could be identified. The respective IS elements and transposon could be assigned to four different IS families (IS*1*, IS*3*, IS*21* and IS*66*). The predicted phage region is unlikely to represent an active phage. Instead, mobilization of the *stx2i* gene by transposons seems the more plausible explanation. A similarity between the BfR-EC-10366 *stx2i* phage and the *stx2i* phages of the other strains was only observed in a small region around the *stx2i* gene. Strains BfR-EC-18848 and BfR-EC-18946 (both with *stx2i* phage integration site II) show almost identical phage regions (99.94% according to BLASTn). In strain BfR-EC-19518 and strain BfR-EC-18545 (representative for IS2-interrupted *stxA2i*), where the same *stx2i* phage integration site (III) was observed, differences in the phage regions were detected due to the presence of additional gene segments in BfR-EC-19518 (Fig. 4)

**Fig. 4.**
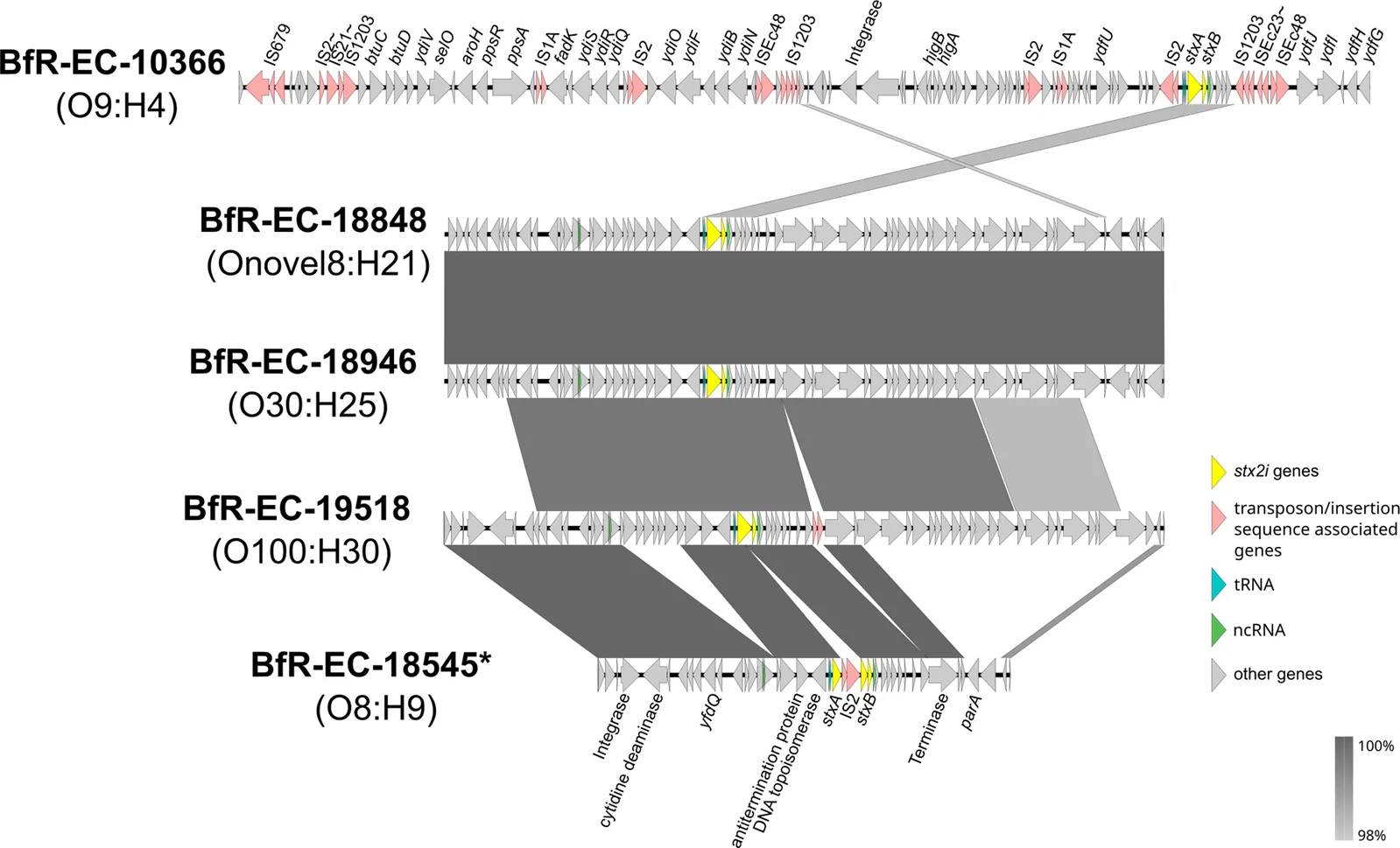
Comparison of *stx2i* phage regions predicted by PHASTER. Genome regions were visualized in Easyfig, and the figure was finalized in Inkscape version 1.3. Strains with a transposon-interrupted *stxA2i* subunit are marked with an asterisk (*). Yellow: *stx* gene subunits A and B; blue: tRNAs; green: ncRNA; red: IS elements; greyscale: sequence identity.

### *Stx2i* subtyping PCR

We designed a new PCR which specifically detects *stx2i* genes and can be integrated into a routine diagnostic workflow to avoid misidentification of *stx2* gene variants. The developed PCR was tested against food and animal strains published here and all known *stx2* subtypes, comprising field strains, as well as toxoid strains [34]. The PCR is resulting in a 640 bp fragment for complete *stx2i* genes and is also able to identify strains with the transposase insertion (Fig. 5). In the case of the IS2 transposon integration, the *stx2i* PCR results in a 1,975 bp fragment. None of the non-*stx2i* strains tested here showed an amplification product in this PCR (Table S3).

**Fig. 5.**
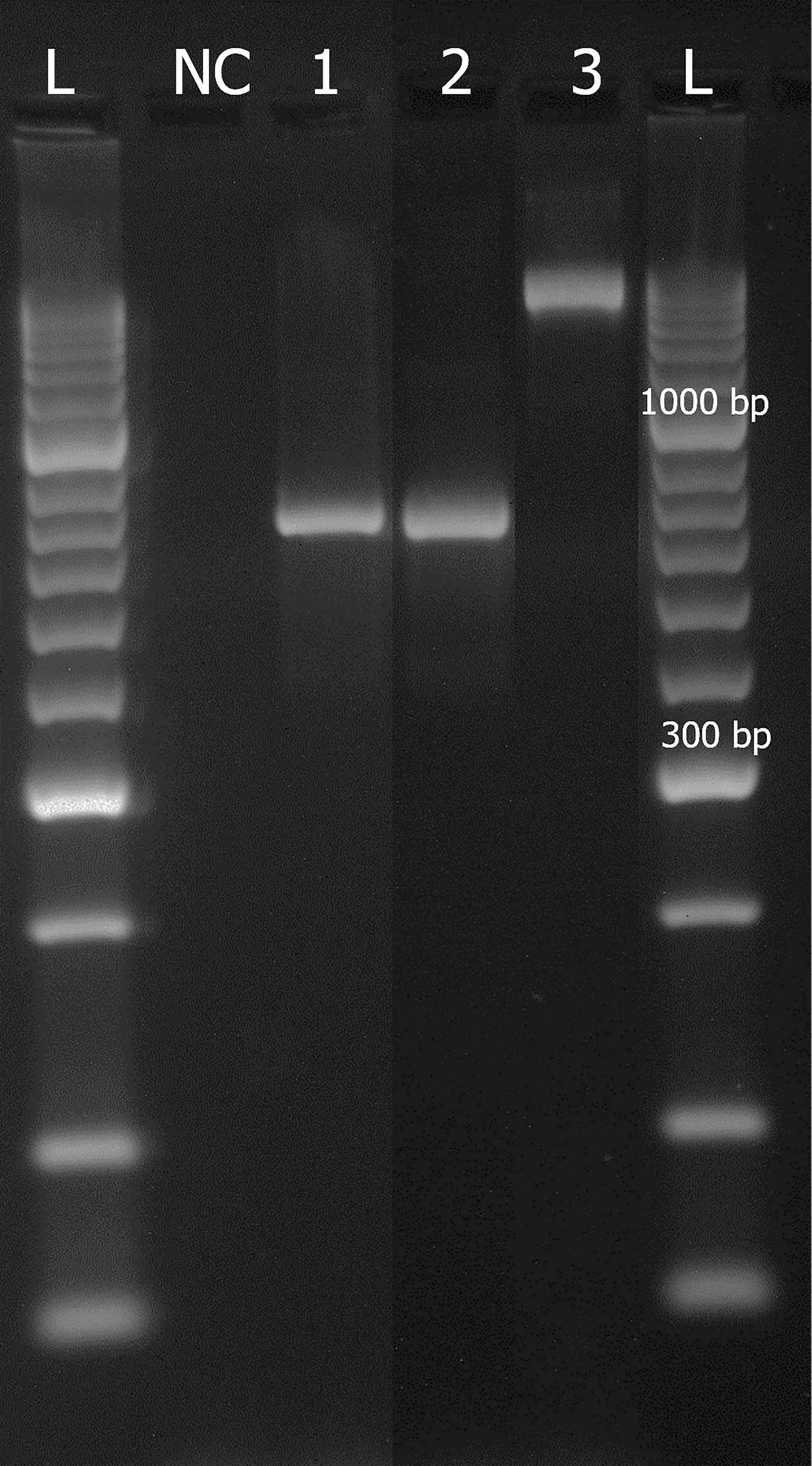
Exemplary results of the newly developed *stx2i* PCR [L: HyperLadder 50 bp; NC: negative control (non-*stx2i* strain); 1: BfR-EC-10366 – CB10366-*stx2i* variant; 2: BfR-EC-20407-663_811_23-*stx2i* variant; 3: BfR-EC-19621 – *stx2i* with insertion].

Newly identified *stx2* variants are a challenge for routine diagnostics [10, 11, 30–32]. There is a variety of PCR methods for STEC subtyping available, and PCR primer and probes originally designed for a certain subtype possibly now show cross-reactions to the new variants due to sequence identities in the binding sites which lead to false interpretation of *stx2* subtypes. We would also like to recommend to reanalyse suspicious strains based on the current knowledge about *stx2i*-positive STEC and other new *stx2* gene variants.

## Conclusion

Taken together, we report STEC strains from food, animals and humans in Germany harbouring complete *stx2i* genes but also inactive *stx2i* operons. Strains with the *stx2i* variant 663_811_23 were isolated from meat of lamb or sheep, of which two food samples were imported meat. This indicates that *stx2i* occurs continuously in more different food matrix types than previously assumed, and due to complex global food chains, the origin of the *stx2i* variant remains unclear, as the 663_811_23 variant was previously isolated from an oyster [2]. A further spread of these strains is likely, and they can also be transmitted to humans.

Genome rearrangements in *E. coli* are very common, and the spread of *stx* phages between strains is also reported [45, 46]. Further analyses of the strains indicated that transposon insertions could be a driver in the evolution of *stx2i*. Pigs might be a host for *stx2* evolution, as all strains harbouring inactive operons were isolated from pigs. However, the inactive variant was associated with a specific genoserotype, suggesting a clonal spread, whereas the active *stx2i* variant was found in STEC of various genoserotypes. The newly developed *stx2i* PCR allows for the discrimination of the *stx2i* gene which is important for routine diagnostics and epidemiological studies on STEC infections.

## Supplementary material

10.1099/mgen.0.001841 Supplementary Material 1.

10.1099/mgen.0.001841 Supplementary Material 2.

10.1099/mgen.0.001841 Supplementary Material 3.

## Abbreviations

NRL-*E. coli*: National Reference Laboratory for *Escherichia coli* including VTEC
STEC: Shiga toxin-producing *Escherichia coli*
Stx: Shiga toxin
