# Supplementary material for: Genomic structure of stx2i genes and prophages determined in Shiga toxin-producing Escherichia coli isolated from food, animal and human samples in Germany
Source: Microb Genom. 2026 Sep 30;12(9):001841. doi: 10.1099/mgen.0.001841 (PMC13641118; doi:10.1099/mgen.0.001841)
Supplement: Supplementary file 1 [file mgen-12-01841-s001.pdf]

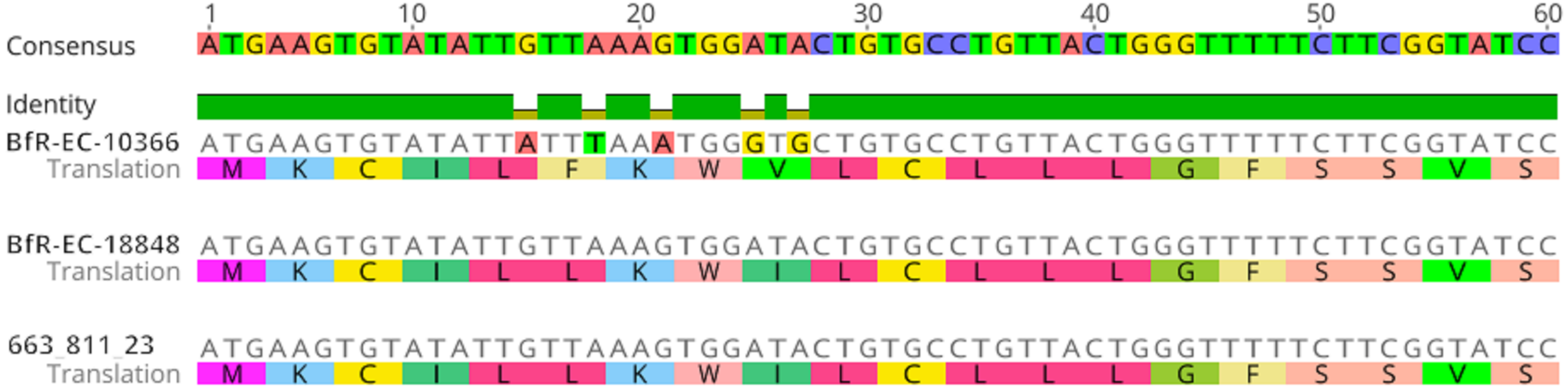
1 10 20 30 40 50 60
Consensus ATGAAGTGTATATTGTTAAAGTGGATACTGTGCCTGTTACTGGGTTTTTCTTCGGTATCC
Identity
BfR-EC-10366 ATGAAGTGTATATTATTTAAATGGGTGCTGTGCCTGTTACTGGGTTTTTCTTCGGTATCC
Translation M K C I L F K W V L C L L L G F S S V S
BfR-EC-18848 ATGAAGTGTATATTGTTAAAGTGGATACTGTGCCTGTTACTGGGTTTTTCTTCGGTATCC
Translation M K C I L L K W I L C L L L G F S S V S
663_811_23 ATGAAGTGTATATTGTTAAAGTGGATACTGTGCCTGTTACTGGGTTTTTCTTCGGTATCC
Translation M K C I L L K W I L C L L L G F S S V S
